# Delays in Diagnosis of Pulmonary Lymphangitic Carcinomatosis due to Benign Presentation

**DOI:** 10.1155/2020/4150924

**Published:** 2020-11-24

**Authors:** Swati Pandey, Shishir Ojha

**Affiliations:** ^1^Elkhart General Hospital, 600 East Blvd, Elkhart, IN 46514, USA; ^2^Elkhart General Hospital, Beacon Medical Group Pulmonology, 500 Arcade Avenue, Suite 210, Elkhart, IN 46514, USA

## Abstract

The diagnosis of lymphangitic carcinomatosis is challenging due to the manifestation of nonspecific symptoms and radiographic abnormalities that bear similarity to those of interstitial lung disease. Herein, we report the case of a 53-year-old woman diagnosed with lymphangitic carcinomatosis from metastatic gastric adenocarcinoma, 3 months after her initial presentation.

## 1. Case

A 53-year-old female was referred to the pulmonary clinic due to cough and chest pressure for the past 3 months, during which several chest X-rays and a computed tomography (CT) scan of the chest were taken. She was initially expectorating green sputum; however, the sputum production reduced after she received antibiotics from her primary care physician (PCP). Additionally, she received two courses of steroids from visits to the emergency department, which stabilized her cough. The patient also had occasional symptoms of postprandial bloating.

**Figure 1:**
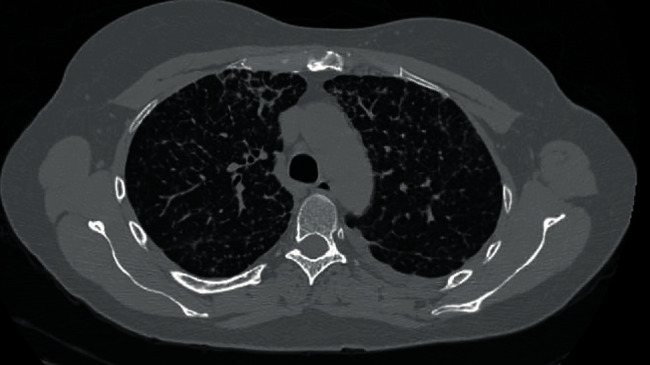
Chest CT scan.

She was a lifelong nonsmoker, who worked in farms and denied any exposure to molds or pets. During examination, she was afebrile with an oxygen saturation of 87% on room air inhalation. Her physical exam revealed coarse breath sounds on auscultation and decreased breath sounds at the level of the left lung base posteriorly. A CT scan of her chest (Figure 1) showed bilateral linear coarse reticulations with no peripheral predilection or ground-glass opacities and a small amount of left pleural effusion; the abdomen did not show any abnormality in the CT scan. Based on her symptoms and chest CT findings, we suspected a diagnosis of interstitial lung disease (ILD).

Laboratory findings were negative for antinuclear antibodies and antineutrophil cytoplasmic antibodies. Video-assisted thoracic surgical biopsy and transbronchial cryobiopsy were discussed, and the risks and benefits of each procedure were explained. She chose to undergo a transbronchial cryobiopsy, which was performed 3 months after her initial presentation to PCP. Three cryobiopsy samples were obtained—two from the lateral basal segment and one from the posterior basal segment of the right lower lobe—for pathologic evaluation. Additionally, four forceps biopsy samples were collected—two each from anterior basal and superior segment of the right lower lobe—for microbiological evaluation. The pathology samples were sent to a tertiary care center to be reviewed by a pulmonary pathologist with expertise in ILD.

**Figure 2:**
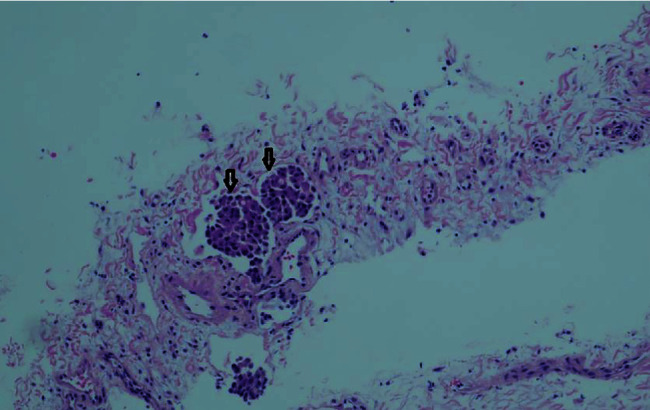
Hematoxylin and eosin-stained slide of cryobiopsy of lung. Arrow pointing to lymph-vascular invasion of tumor cells.

**Figure 3:**
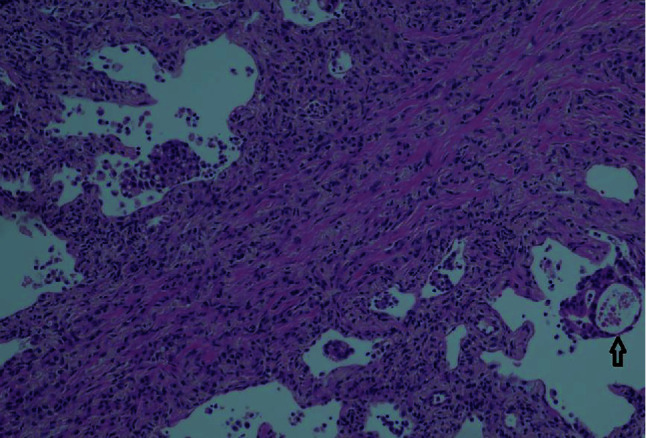
Hematoxylin and eosin-stained slide of cryobiopsy of lung. Arrow pointing to adenocarcinoma.

Based on the morphology, the patient was diagnosed with a tumor that was characterized as metastatic adenocarcinoma with lymphangitic spread (Figures 2 and 3). Tumor cells were positive for keratin and negative for CD31, thus confirming the diagnosis. A second round of histological staining was negative for thyroid transcription factor-1, GATA 3, mammaglobin, and gross cystic disease fluid protein-15 (GCDFP-15), thereby excluding the lungs and breast as primaries. As CDX2 was strongly positive, gastric primary was suspected. The differential diagnosis in the upper gastrointestinal primary or pancreatobiliary primary at this stage of staining supported the assumption.

**Figure 4:**
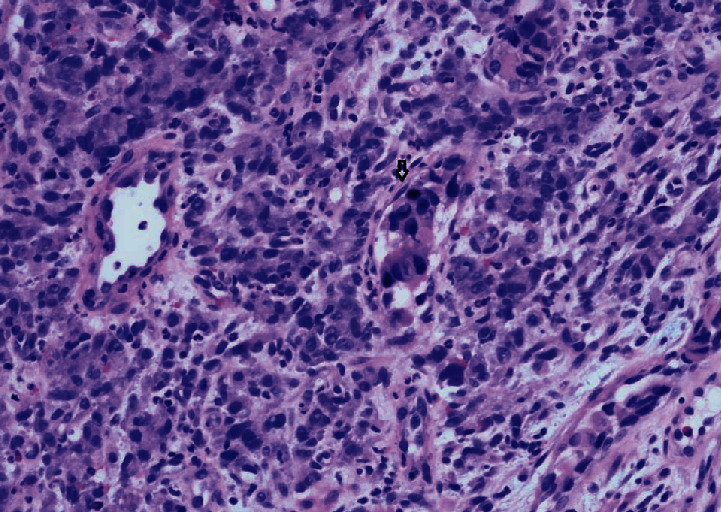
Hematoxylin and eosin-stained slide of Gastric Biopsy: arrow pointing to gastric adenocarcinoma.

Thereafter, the patient underwent a repeat CT scan of the abdomen and pelvis, which did not reveal any mass or abnormality to suggest a primary. Three weeks after the bronchoscopic biopsy, she underwent an upper endoscopy, which revealed an ulcerated gastric mass occupying the cardia and antrum of the stomach. Biopsies from the mass revealed moderate to poorly differentiated invasive adenocarcinoma with lymphovascular invasion (Figure 4).

Two days after the endoscopic biopsies, the patient was seen by an oncologist in an outpatient setting where several chemotherapy options ranging from aggressive regimens, such as FOLFOX, to the least aggressive 5 FU with leucovorin were discussed. However, the patient declined the treatment. Subsequently, she was hospitalized three times, 2 weeks apart, for worsening dyspnea and was treated with thoracentesis on each occasion. During her last admissions, a chest tube was placed due to iatrogenic pneumothorax after thoracentesis. The patient experienced worsening dyspnea and hypoxemia, and comfort measures were initiated. The patient died 10 days after the last hospitalization, about 2 months after her diagnosis.

## 2. Discussion

We report the case of occult gastric malignancy with pulmonary lymphangitic carcinomatosis (PLC).

Pulmonary metastasis is rare with gastric cancer, representing less than one percent of distant metastasis. The most commonly seen pattern of pulmonary metastasis is hematogenous which accounts for 52.3%, followed by pleural metastasis (35.2%), and lymphangitic spread is the least common of this rare pattern, accounting for only 26.4% of pulmonary metastasis [1]. Gastric lesions or abnormality in other viscera were absent on the CT scan of the abdomen. Her chest CT revealed interlobular septal thickening. The absence of mediastinal or hilar lymph node enlargement and lung masses or nodules excluded a diagnosis of malignancy. Despite widespread pulmonary parenchymal abnormalities, there was no lymph node involvement. Mediastinal or hilar lymphadenopathy is commonly seen with lymphangitic spread [2], but lymph node involvement is not essential. The mechanism of lymphangitic spread is hematogenous tumor embolism to the lungs and rarely due to contiguous lymphangitic spread [3].

The diagnosis of metastatic cancer with pulmonary carcinomatosis is often delayed by months from the onset of symptoms and imaging studies. This delay is mainly due to two attributes that are strongly associated with its presentation and imaging studies. First, patient characteristics play an important role. Most patients present with dry cough and dyspnea due to pulmonary parenchymal involvement, irrespective of the origin of the primary. Hence, the workup for the symptoms is performed and does not involve imaging or diagnostic tests, which can reveal the primary. A patient's age is usually less than that of a typical lung cancer patient because a wide variety of cancers that occur at a young age may present with lymphangitic spread. Patients are often nonsmokers, thereby lowering the suspicion for lung cancer. Moreover, patients are often empirically treated with antibiotics for presumed respiratory tract infection and inhalers for dyspnea.

Second, a radiologist is often misled by imaging patterns, whether on chest X-ray or chest CT, and the requesting physician may be convinced of an ILD diagnosis. The CT pattern of PLC is very similar to that of many ILDs, including sarcoidosis, which is an arduous task to differentiate. Subtle differences do exist, but these are not easy to spot and include a great involvement of the interlobular septa and interstitium in PLC and more distortion of secondary pulmonary lobule due to fibrosis in sarcoid [4]. Thus, owing to the clinical presentation and radiographic patterns, our focus was on the diagnosis of an underlying primary pulmonary parenchymal disease, specifically an ILD, rather than casting a wider net, which would have included an examination for malignancy.

We performed a literature search using the PubMed database for studies reporting cases of lymphangitic carcinomatosis that were suspected as an ILD by treating physicians. The extracted publications are listed in Table 1 [5–18]. Information on the types of delays that occurred in the case reports are listed (Table 1), which included time interval from the first occurrence of a symptom to the first contact with a physician and the interval from symptoms onset, diagnosis, and presentation to death.

Delays in the diagnosis of primary lung cancer diagnosis range from 7 days to 6 months and from onset of symptoms to contact with a physician [19]. The largest trial that has investigated 380 consecutive patients with primary lung cancer found that the median duration from the onset of symptoms to visit with a physician was 7 days, that from the physician's visit to diagnosis was 31 days, and that from symptom onset to diagnosis was 50 days [20]. As depicted, the wait times are similar to the delays in the diagnosis of primary lung cancer.

The reason for the delay in the diagnosis of PLC can be due to the misinterpretation of the presentation and radiographic abnormality by the treating physician, and such delay reduces patients' duration of survival after diagnosis. Compounding this grave situation is that patients spend their remaining life under intensive care while undergoing invasive procedures to diagnose what was misinterpreted as ILD. The overall 5-year survival rate for lung cancer for all stages is 19.4% [21]. In comparison, as depicted in the table, several diagnoses of PLC occurred in the intensive care unit on vented patients, and most of them died within a day to few weeks thereafter.

The misinterpretation of PLC as an ILD is more likely to occur if the imaging study shows findings of only pulmonary carcinomatosis, i.e., only interstitial changes, which are very similar to many ILDs, without lung mass or a nodule. Although many ILDs present with lymph node enlargement, the presence of mediastinal or hilar lymph node enlargement raises the suspicion of malignancy.

The patient in this case report did not have mediastinal or hilar lymphadenopathy, unlike most patients with pulmonary carcinomatosis who have lymphadenopathy. If mediastinal or hilar lymphadenopathy were present, then a much more conservative approach, such as biopsy of the lymph node with endobronchial ultrasound with or without transbronchial forceps biopsy, would have been considered. Instead, due to the presentation, a much more invasive approach than needed for the diagnosis of malignancy, such as transbronchial cryobiopsy, was undertaken. Transbronchial cryobiopsy is a new technique that enables pulmonologists to obtain a larger specimen than the traditional transbronchial forceps biopsy [22], but whether it can serve as an alternative to surgical biopsy for the diagnosis of ILDs is a subject of investigation. Nevertheless, the risks of pneumothorax and bleeding are higher than those of forceps biopsy [23], but the overall morbidity is lower than that of surgical lung biopsy [23]. Therefore, patients with PLC undergo more invasive biopsies than needed to obtain a diagnosis, such as surgical lung biopsies, due to the resemblance to ILD in CT scans. However, the limited therapeutic options available for ILDs prevent many patients from undergoing a diagnostic biopsy as the diagnostic procedures are considered to be too risky by their pulmonologist [24]. Therefore, this practice can place patients at the risk of not getting diagnosed for a lethal condition while getting empirically treated for ILD.

ILD is more prevalent than PLC. Therefore, due to the similarity in presentation, clinicians will first seek and investigate the diagnosis of ILD [25] [26], hence being misled. Interestingly, the misdiagnosis of PLC occurs with an even less common disease entity, i.e., Erdheim–Chester disease, which is presented as PLC [27]. Erdheim–Chester disease is a rare disease listed under National Organization for Rare Disorders. Thus, there is precedence for the misdiagnosis of PLC, not only with ILD but also other conditions, warranting robust investigations to enable definite conclusions to be made.

## Figures and Tables

**Table 1:** Case reports of PLC suspected as an ILD.

S. NO	1	2	3	4	5	6	7	8	9	10	11	12	13	14
Age/gender	15 F	7 M	31 F	45 F	53 F	63 M	62 F	62 M	39 F	24 M	30 M	30 M	25 M	62 M
Symptoms	Cough, sob, lethargy, satiety, anorexia	Respiratory distress	Dry cough, dyspnea	Dry cough	Productive cough and dyspnea	Progressive dyspnea and cough	Progressive dyspnea, weight loss, and rash.	Worsening dyspnea	Dyspnea, dry cough.	Dry cough, night sweats, dyspnea hemoptysis	Dry cough, night sweats, dyspnea on exertion, weight loss	Cough, sob, fever, and chest pain.	Cough, dyspnea on exertion	Dyspnea, dry cough fever for six weeks
Chest CT	Thickened interlobular septa	Mediastinal lymphadenopathy, B/l interstitial infiltrates	Mediastinal lymphadenopathy. Thickened interlobular septa, ground-glass opacities	Interlobular septal thickening, scattered GGO, nodularity along fissures,	Interstitial thickening, crazy paving, mediastinal lymphadenopathy	Enlarged med LN, diffuse reticular thickening, reticular-nodular pattern.	Diffuse reticulonodular and ground-glass opacities, pleural effusions	Subpleural reticulations pulmonary nodules, enlarged mediastinal LN	Interstitial and ground-glass opacities. Borderline mediastinal lymphadenopathy	Ground glass opacities, diffusely thickened interlobular septa, mediastinal and hilar LN enlarged	Thickening of peribronchovascular, interstitial, and interlobular septa. Ground glass opacities	Mediastinal lymphadenopathy and reticulonodular interstitial pattern.	Diffuse interstitial prominence	Bilateral interstitial infiltrates
Diagnostics	Open lung biopsy	Open lung biopsy	Transbronchial biopsy	Open lung biopsy	Bronchoalveolar lavage	Transbronchial biopsy	Bronchoalveolar lavage negative, cervical skin biopsy positive	Bronchoalveolar lavage and transbronchial biopsy	VATS surgical biopsy	Bronchoalveolar lavage. Central bronchoscopic biopsies. Gastric origin by EGD.	Bronchoalveolar lavage showed atypical cells. Transbronchial biopsies could not be done.	Bronchoscopy could not be completed. Diagnosed by surgical lung biopsy	Open lung biopsy	Transbronchial biopsy, prostate biopsy
Diagnosis	Adenocarcinoma unknown primary	Renal adenocarcinoma	Adenocarcinoma of colon	Signet ring gastric adenocarcinoma	Melanoma	Signet ring gastric adenocarcinoma	Signet ring gastric adenocarcinoma	Pulmonary adenocarcinoma	Gastric adenocarcinoma	Gastric adenocarcinoma with focal signet ring.	Signet ring cell gastric adenocarcinoma on EGD biopsy	Pulmonary adenocarcinoma	Pulmonary adenocarcinoma	Prostate adenocarcinoma
Symptom onset to diagnosis (days)	107	NA	180	117	Over 30 days	150	180	NA	NA	60	60	NA	379	42
Symptom to death (days)	108	NA	191	119	NA	159	180	NA	NA	NA	NA	NA	382	NA
Presentation to death (days)	70	NA	16	29	NA	NA	67	NA	NA	NA	NA	NA	17	NA
Diagnosis to death (days)	1	NA	11	12	NA	7	7	NA	NA	NA	NA	NA	3	NA
Therapy prior to diagnosis	Prednisone, clarithromycin, salbutamol, antitubercular, antipneumocystis therapy.	NA	Steroids for sarcoidosis. Folfox after diagnosis	Ciprofloxacin and erythromycin for bronchitis, right heart Cath, sildenafil, and ambrisentan for Pulm HTN.	Vancomycin and piperacillin-tazobactam	Antibiotics and steroids	Immunosuppressive therapy	NA	Steroids	Broad-spectrum antibiotics for 14 days, high dose steroids	Piperacillin-tazobactam, ciprofloxacin, and oseltamivir	NA	Antibiotics.	Not applicable. Survived.
Setting of diagnosis	ICU vent	NA	Post biopsy, ICU, vent	ICU. Biopsy while intubated, palliative postdiagnosis	Non-ICU setting	ICU on vent support	NA	Not in ICU	NA	NA	Na	NA	ICU	NA
Metastasis	Not available (NA)	NA	Thoracic and lumbar spine	Abdominal wall, tibia, femur, right humerus, lumbar spine	NA	NA	Skin	Contralateral pulmonary and spinal metastasis.	NA	Lung, pleura, right ribs, right iliac bone	NA	NA	NA	NA
Author	Gilchrist et al., Eur Resp Review [5]	Vanclaire et al., Arch Fr Pediatrics [6]	Thomas and Lenox, CMAJ [7]	Khachekian et al., J Am Osteopath Assoc. [8]	Biswas et al., Am Journal of Medicine [9]	Dikis et al., Clinics in Surgery [10]	Wang et al., Poster, chest meeting [11]	Guler et al. [12], Journal of clinical respiratory disease & care.	Gleason et al., J of clinical and diagnostic research [13]	Moubax et al., BMC research notes [14]	Meltem et al., Turkish thoracic journal [15]	Blanco et al., An Med Interna [16]	Mapel et al., Lung Cancer [17]	Cohen et al. [18], Respiration
